# Practical Medicine

**Published:** 1877-10

**Authors:** 


### III. Practical Medicine.

The Tenacity of the Life of Tape-worms.—Prof. Edward Perroneito.—(*The Boston Medical and Surgical Journal*, September 13, 1877.) In order to decide the important question of the tenacity of life of tape-worms and their larval forms, Prof. Perroneito made a long series of experiments and observations on *cysticercus cellulosæ*, and was enabled to ascertain exactly the lowest temperature requisite to insure the death of cysticerci and other animal parasites. In carrying on these investigations,he employed Schultze's heating table with neutral tincture of carmine and hæmatoxiline. His method depends (a) on the fact that a cysticercus keeps moving actively about while the temperature of the fluid in which it is placed is gradually raised; if one of these worms freshly prepared be placed in pure water, or in a weak solution of common salt, which is then gradually heated up to the temperature of warm-blooded animals, or still higher, the parasite twists and turns around with considerable activity, using its suckers and proboscis as organs of locomotion; (b) on the greater readiness with which dead tissues are stained by coloring fluids than are the same tissues during life. Experiments were made to ascertain the value of these two means of diagnosis above described. If the cysticercus freshly taken from a pig be prepared in the manner just mentioned, placed on a Schultze's warming slide and examined under the microscope, it was found that it began to move in most cases when the temperature reached 30° or 35° C. As the temperature rose still higher, the activity of the animal increased, especially while passing from 38° to 49° C. As the temperature gradually increased, the movements of cysticercus cellulosæ cease occasionally at 45°—46° C, sometimes at 47°, more frequently at 48° C, and out of over fifty experiments in only one case did the cysticercus keep up its motions beyond 49°, ceasing then, however, at 50° C.

As soon as its movements end the parasite is dead, and can not be restored by again lowering the temperature to that of the surrounding air and raising it a second time. At none of the intermediate temperatures does the cysticercus show the least signs of life. But a more convincing proof of the death of the parasite is obtained from the use of staining fluids, which color the *dead*, but not the living tissues of tape-worms.

A living cysticercus with its head everted may be put in neutral tincture of carmine, or in hæmatoxiline for from two to twelve hours or more without being colored; the staining begins only when the cysticercus dies. Therefore if the cysticercus is first brought to a temperature high enough to kill it, that is 50° C, and then left in one of the above mentioned tinctures, it colors intensely in less than three-quarters of an hour.

The staining begins at the head and proceeds towards the extremities of the caudal cyst. The head colors more deeply and rapidly than the neck, on account of the calcareous bodies which are less numerous in the remaining parts of the body. If a cysticercus from the pig be brought gradually to a temperature of 50° C, and then swallowed alone, or with butter or a crumb of bread, it never produces a tænia.

Some courageous students who volunteered to try the experiment did so without evil result. Prof. Perroneito's investigations were repeated upon other forms of worms, and the results were always the same. The cysticercus of the pig can seldom survive a temperature of 49° C, and exposure to the temperature of 50° C, for more than a minute leads to its death. The trichina spiralis, either free or incysted, as found by several experiments always died at 48° C. These experiments have a great scientific and practical value, assuring on the one hand the highest temperature which cysticercus trichina and other similar parasites can endure. The tenacity of life generally attributed to the helminths and larvæ of like forms is, therefore, much less than usually supposed. These experiments also show the harmlessness of flesh infested by these parasites, when it has once been raised to a temperature above $50^0$ C, although it may remain there not longer than five minutes.

